# Environmental and genetic control of cold tolerance in the Glanville fritillary butterfly

**DOI:** 10.1111/jeb.13247

**Published:** 2018-03-03

**Authors:** M. A. de Jong, M. Saastamoinen

**Affiliations:** ^1^ School of Biological Sciences University of Bristol Bristol UK; ^2^ Organismal and Evolutionary Biology Research Programme University of Helsinki Helsinki Finland

**Keywords:** candidate genes, chill coma recovery, developmental plasticity, *flightin*, genetics, insects, Lepidoptera, physiology, thermal acclimation

## Abstract

Thermal tolerance has a major effect on individual fitness and species distributions and can be determined by genetic variation and phenotypic plasticity. We investigate the effects of developmental and adult thermal conditions on cold tolerance, measured as chill coma recovery (CCR) time, during the early and late adult stage in the Glanville fritillary butterfly. We also investigate the genetic basis of cold tolerance by associating CCR variation with polymorphisms in candidate genes that have a known role in insect physiology. Our results demonstrate that a cooler developmental temperature leads to reduced cold tolerance in the early adult stage, whereas cooler conditions during the adult stage lead to increased cold tolerance. This suggests that adult acclimation, but not developmental plasticity, of adult cold tolerance is adaptive. This could be explained by the ecological conditions the Glanville fritillary experiences in the field, where temperature during early summer, but not spring, is predictive of thermal conditions during the butterfly's flight season. In addition, an amino acid polymorphism (Ala‐Glu) in the gene *flightin*, which has a known function in insect flight and locomotion, was associated with CCR. These amino acids have distinct biochemical properties and may thus affect protein function and/or structure. To our knowledge, our study is the first to link genetic variation in *flightin* to cold tolerance, or thermal adaptation in general.

## Introduction

Temperature is one of the main environmental factors determining species' distributions and driving local adaptation (Sinclair *et al*., 2003; Angilletta, 2009). In ectothermic species such as insects, variation in temperature directly affects core physiological processes and consequently has major effects on behaviour, life history and fitness (Gilchrist & Partridge, 1999; Huey & Berrigan, 2001; Angilletta, 2009). Both genetic variation and phenotypic plasticity, as well as their interaction (i.e. genetic variation in the plasticity response, or G × E), can contribute to variation in thermal tolerance within and among species (Hoffmann *et al*., 2005; Angilletta, 2009; Chown *et al*., 2010). Understanding how organisms cope with temperature extremes, either through phenotypic plasticity, genetic change or their interaction, is particularly relevant for predicting the effects of climate change on species distributions, as climate models forecast increased variation in temperatures, reduced predictability of this variation and novel thermal extremes (Gienapp *et al*., 2008; Hoffmann & Sgro, 2011; Vasseur *et al*., 2014; Sgro *et al*., 2016).

Plasticity of the physiological response to temperature can be an important adaptive strategy for organisms in coping with changing thermal conditions (Terblanche & Chown, 2006; Angilletta, 2009). Alternatively, thermally induced plasticity can be a nonadaptive response caused by physiological sensitivity or stress in response to environmental variation (Leroi *et al*., 1994; Deere & Chown, 2006). Insects often show distinct physiological differences among life stages, during which they may experience very different ecological conditions, so selective pressures on thermal physiology might vary among life stages. Developmental plasticity can be adaptive when conditions experienced during development reliably predict the environmental conditions the organism will encounter later on in life (predictive adaptive response, Monaghan, 2008; van den Heuvel *et al*., 2013). Thermal plasticity during the adult stage, or adult thermal acclimation, can enable reversible short‐term adjustments in physiology to temperature fluctuations. Although physiological responses and their plasticity, to thermal conditions, are well studied in insects and other ectotherms, few studies have compared the phenotypic effects of developmental and adult plasticity on thermal tolerance (but see Colinet & Hoffmann, 2012; Marais & Chown, 2008; Zeilstra & Fischer, 2005).

Studying the genetic basis of thermal tolerance is key to understanding adaptation to thermal conditions and thermal limits to species' distributions (Franks & Hoffmann, 2012). Although knowledge of the genetic control of thermal adaptation remains limited, a number of functional gene groups have been linked to variation in thermal tolerance, either through differential gene expression or genetic variation underlying structural protein changes. These include, for instance, stress response genes such as heat shock proteins (Frydenberg *et al*., 2003; Sørensen *et al*., 2003), and metabolic genes coding for enzymes involved in core metabolic processes (Morgan & Mackay, 2006; de Jong *et al*., 2013). In insects, most progress on the genetics of thermal adaptation has been made with studies on *Drosophila* (e.g. Hoffmann *et al*., 2003, 2005; Morgan & Mackay, 2006), but much less is known about other insect groups.

When studying thermal tolerance, it is important to take into account the ecological conditions that shape thermal adaptation in nature. The well‐studied Glanville fritillary metapopulation in the Åland Islands in Finland lies at the northern limit of the species' range, where adaptation to cold thermal conditions is critically important for individual performance and population dynamics (Saastamoinen, 2007; Ojanen *et al*., 2013). Previous Glanville fritillary studies have linked sequence and expression variation in several candidate genes to thermal physiology and fitness (Kvist *et al*., 2013; Saastamoinen *et al*., 2013a; Luo *et al*., 2014) and have shown that early‐life conditions can have important effects on performance and fitness later in life (Kallioniemi & Hanski, 2011; Saastamoinen *et al*., 2013b).

In this study, we tested the effects of thermal conditions during different life stages on cold tolerance in the Glanville fritillary. We measured chill coma recovery (CCR), a well‐established proxy for cold tolerance (Macmillan & Sinclair, 2011), at the beginning of the adult stage following two temperature treatments during late larval and pupal development. We then tested the effects of both thermal treatments during the adult stage on late adult CCR. We hypothesize that a cooler developmental temperature will result in increased cold tolerance (a faster recovery time) in early adults and that this effect is reversible through acclimation to opposite thermal conditions during the adult stage. In addition, we investigate the genetic basis of cold tolerance by associating CCR with SNP variation in a set of candidate genes that were selected on the basis of previous genomics and transcriptomics studies in the same species, which have a known role in insect physiology. We discuss our findings in the context of the well‐known ecology of the Glanville fritillary in the Åland Islands.

## Materials and methods

### Study material and pre‐experiment rearing

We reared wild‐originating butterflies in the laboratory for three generations to minimize cross‐generational and early‐life effects of environmental variation. The great‐grandparents of the larvae used in the experiment were collected as fifth instar larvae in the autumn from 26 relatively large local populations that are part of a large network of about 4000 habitat patches in the Åland Islands in Southwest Finland. Previous genetic analyses have revealed a relatively high genetic differentiation (*F*_ST_ = 0.1; Saccheri *et al*., 2004) within the large population network, yet genetic clustering occurs on a spatial scale larger than individual local populations (Orsini *et al*. 2008). Inbreeding can locally be high but primarily in very small and isolated local populations, that is not those included here (Saccheri *et al*. 1998). Larvae were first kept in diapause at 4 °C and 70% humidity for 6 months and then reared under 12 : 12‐h light/dark photoperiod and 28 : 15 °C day/night temperature cycle (standard larval rearing conditions in this study system, see, e.g. Saastamoinen *et al*., 2013a,b). Larvae were fed with fresh leaves of greenhouse‐grown *Plantago lanceolata* (the common host plant for this species in the Åland Islands). The butterflies were kept in soft netting cages (diameter 20 cm) with a maximum of 10 individuals per cage and provided with sponges soaked with a 20% honey water solution as *ad libitum* food source. The butterflies mated in the cages, whereas it was ensured there were no within‐family matings. The following generations were reared under the same conditions, including 6‐month diapause periods. The third generation was used for the experiment and consisted of 18 full‐sib families (3–18 sibs per family) split into two temperature treatments from the final instar onwards. We restricted our experimental treatments to the final instar and pupal stage of development, as wild *Melitaea cinxia* caterpillars live gregariously in all but the final instar. Rearing them individually at earlier stages would increase mortality, thereby reducing the sample size and potentially affecting the measured traits (Rosa *et al*., 2017).

### Experimental treatments and phenotypic measurements

Final instar larvae were randomly assigned to two thermal rearing conditions in climate‐controlled cabinets (Sanyo MLR‐351), in which they were individually reared to the adult stage in small transparent plastic containers (diameter 6 cm). We used the following two thermal treatments: ‘cool’ with a 22 : 10 °C day/night temperature cycle and a 12 : 12‐h light/dark photoperiod; and ‘warm’ with a 28 : 18 °C day/night temperature cycle and a 12 : 12‐h light/dark photoperiod. These temperatures were based on the natural range of spring and summer temperatures in Åland and take into account that – due to their dark colour and basking behaviour – larvae reach far higher body temperatures than the prevailing ambient temperatures in the spring (up to 15 °C higher in sunny conditions, unpublished data). As a proxy for adult size, pupal weight was measured to the nearest 0.1 mg 1 day after pupation.

The butterflies were measured for CCR on the first day after eclosion (CCR1) and again on the 6th day after eclosion (CCR6). On the day of eclosion, the containers with the butterflies were transferred at 5 pm to a climate‐controlled room with 23 : 18 °C, 12 : 12‐h day/night temperatures (standard conditions for adult *M. cinxia* in the laboratory). We chose to expose the butterflies from both temperature treatments to the same standard conditions just before the experiment because we were specifically interested in detecting the persisting longer‐term effects resulting from the different acclimation treatments and not the possible short‐term effects of different adult temperatures just before CCR measurement. The next day at 9 am, the containers were placed in an environmental chamber (WTC Binder) at −5 °C for 2 h. This approach of rapid cooling has the benefit of limiting short‐term cold hardening responses that can occur with a more gradual ramping of the temperature change (Overgaard *et al*., 2012). After this, the containers were taken out of the freezer in a climate‐controlled room at 21 °C. Individual CCR was measured as the time it took (in seconds) for the butterfly to recover from lying flat to standing on its legs. The room temperature was recorded to the nearest 0.1 °C at the start and the end of the CCR measurement. After the CCR1 measurement, the butterflies were randomly assigned to the ‘cool’ and ‘warm’ thermal treatment cabinets. The butterflies were individually marked with a number written on the hind wing with permanent marker and kept as described above but with males and females in separate cages. CCR6 was measured after 5 days as described above. The butterflies were then stored at −80 °C until DNA extraction.

### Genotyping

DNA was extracted from thorax tissue using a Nucleo Spin 96 Tissue kit (Macherey‐Nagel GmbH & Co. KG, Duren, Germany) following the manufacturer's protocol.

The candidate genes and SNPs were selected on the basis of their known function in insect physiology and previous Glanville fritillary studies, including genome analysis (Ahola *et al*., 2014), genetic association studies (Orsini *et al*., 2009; Saastamoinen *et al*., 2013a; de Jong *et al*., 2014) and gene expression studies (Kvist *et al*., 2013). We genotyped the butterflies for 18 SNPs in 11 genes, including SNPs with expected minor allele frequency > 0.2 as estimated from an EST library: *cytochrome P450* (*Cyp337*, 3 SNPs), *flightin* (*fln*, 1 SNP), *glucose‐6‐phosphate dehydrogenase* (*G6PD*, 2 SNPs), three *heat shock protein 70* genes (*Hsp70*, five SNPs in three genes), *JNK interacting protein* (*JNK*, 1 SNP), *peripheral‐type benzodiazepine receptor* (*PBR*, 1 SNP), *phosphoglucose isomerase* (*Pgi*, 3 SNPs, described by Orsini *et al*., 2009), *succinate dehydrogenase complex subunit D* (*SDHD*, 1 SNP) and *troponin‐T* (*TnT*, 1 SNP). A list of the selected markers and their selection criteria is given in Table S1, Appendix S1.

SNP genotyping was performed at the Institute for Molecular Medicine Finland (FIMM, Helsinki, Finland) using Sequenom iPLEX Gold chemistry (Sequenom Inc., San Diego, CA, USA) and validated for seven independent samples by direct genomic sequencing with the ABI 3730 platform (Life Technologies Ltd, Paisley, UK) according to the manufacturer's protocols. We used MassARRAY Assay Design 3.1 (Sequenom Inc.) to design amplification primers and extension probes, whereas the validation primers covering the Sequenom primer sites were designed using Primer3 (Rozen & Skaletsky, 2000). The SNPs were quality‐checked with manual signal cluster evaluation and Hardy–Weinberg equilibrium (HWE) statistics (1 degree of freedom with 5% significance level). Three SNPs in two genes were excluded because they failed to meet our inclusion criteria: one SNP was not polymorphic in this data set, contrary to what had been observed in the EST data, whereas two SNPs showed significant deviations from HWE (also see Table S1, Appendix S1). See also Wong *et al*., 2016 for additional info on SNP selection and genotyping.

### Statistical analysis

We used linear mixed effects models to test for the effects of thermal conditions on CCR times at day 1 (CCR1) and 6 (CCR6) after butterfly eclosion, and to test for associations of candidate SNPs with CCR. All statistical analyses were carried out in R (R Core Team, 2014) using the package lme4 (Bates *et al*., 2012). CCR was log_10_ transformed to improve normality. We analysed the effects of developmental thermal treatment on CCR1 using a model with larval treatment as fixed factors, pupal weight and room temperature as covariates, and family as a random effect to account for relatedness between individuals. To analyse the effects of both developmental and adult thermal treatment on CCR6, we added adult thermal treatment as a fixed factor to the model. We gradually removed interaction terms and main fixed effects and chose the best fitting model based on Akaike's information criterion (AIC, Crawley, 2005). Chi‐square and associated *P*‐values for fixed effects were obtained by comparing the full model with a model excluding the effect using a likelihood ratio test.

To associate candidate SNPs with CCR, we used a model with CCR1 and CCR6 as repeated measures, and the focal SNP as a linear fixed effect, assuming an additive allelic effect. To correct for the effects of the larval and adult thermal treatments, we included these as fixed factors, as well as pupal weight as covariate and family as a random factor. We gradually removed interaction terms of fixed effects based on AIC and obtained chi‐square and associated *P*‐values for fixed effects as above. For the genotype–phenotype association analyses, we applied Bonferroni correction for multiple testing.

## Results

### Phenotypic responses

Table 1 gives the means, standard errors and sample sizes for the measured phenotypic traits, per sex and treatment group. Cooler thermal conditions during the final instar increased larval development time by 4 days in males and 5 days in females (larval treatment; *F*_1,140_ = 102.4, *P *<* *0.0001), with males developing faster than females under both conditions (sex; *F*_1,140_ = 90.7, *P *<* *0.0001). Individuals that developed under cooler environmental conditions also weighed more (larval treatment; *F*_1,140_ = 23.6, *P *<* *0.0001) and had longer pupal development times (larval treatment; *F*_1,140_ = 1141.4, *P* < 0.0001) than those under warm conditions in both sexes. Females weighed more than males (sex; *F*_1,140_ = 98.4, *P* < 0.0001) and tended to have longer pupal development time than males (sex; *F*_1,140_ = 3.9, *P* = 0.05). Interactions between larval treatment and sex were not significant (*P *>* *0.1 for all).

**Table 1:** Trait means and standard errors (SEs) for larval development time (days), pupal development time (days), pupal weight (mg) and chill coma recovery (CCR, in seconds and log_10_ transformed) measured on the first day of the adult stage, are given for the two developmental treatment groups (cool and warm) and for males and females. In addition, the means and SEs are given for CCR (in seconds and log_10_ transformed) measured on the sixth day of the adult stage, for the two adult thermal treatment groups (warm and cool) and for each sex

Trait	Males		Females	
Developmental treatment	Cool (*n* = 49)	Warm (*n* = 64)	Cool (*n* = 15)	Warm (*n* = 12)
Larval dev. time (days)	31.6 ± 0.3	28.4 ± 0.2	36.5 ± 0.7	32.3 ± 1.0
Pupal dev. time (days)	19.5 ± 0.3	11.1 ± 0.1	20.3 ± 0.5	11.6 ± 0.2
Pupal weight (mg)	152.9 ± 2.3	141.6 ± 1.4	181.1 ± 4.8	178.2 ± 6.1
CCR time day 1 (s)	401.8 ± 16.9	314.6 ± 7.7	427.5 ± 24.1	391.4 ± 23.7
Log_10_ CCR time day 1	2.59 ± 0.02	2.49 ± 0.01	2.62 ± 0.02	2.58 ± 0.03

Adult treatment	Cool (*n* = 53)	Warm (*n* = 58)	Cool (*n* = 14)	Warm (*n* = 11)
CCR time day 6 (s)	376.7 ± 14.9	418.4 ± 16.2	432.4 ± 30.7	511.5 ± 37.3
Log_10_ CCR time day 6	2.65 ± 0.02	2.60 ± 0.02	2.62 ± 0.03	2.70 ± 0.03

**Figure 1:**
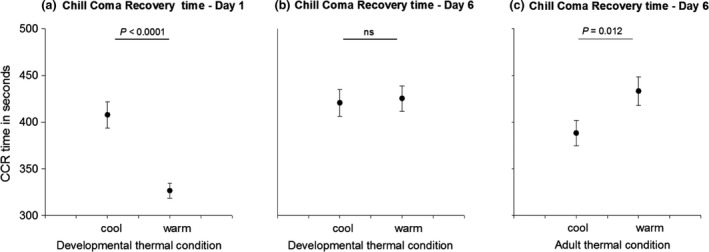
(a) The effect of developmental thermal treatment on mean chill coma recovery (CCR) time of adults on day 1; (b) the effect of developmental thermal treatment on the mean CCR time of adults on day 6; (c) the effect of adult thermal treatment on the mean CCR time of adults on day 6. Error bars represent standard errors.

Chill coma recovery time at day 1 of adult life (CCR1) was significantly influenced by developmental thermal conditions (*P *<* *0.0001, Table 2): individuals that experienced cooler thermal conditions during development took on average 80 s, or 25%, longer to recover from chill coma (Table 1, Fig. 1). Sex, pupal weight or room temperature at the start of the CCR measurement (*T*_START_) did not influence CCR time significantly (*P *>* *0.1 for all, Table 2). The interaction terms were nonsignificant and removed based on the model AIC scores. The random family effect explained 6% of the total variance (family variance = 0.00054 ± 0.023 SD; residual variance = 0.0088).

**Table 2:** Likelihood ratio test results for temperature treatments and other fixed model effects on log_10_ chill coma recovery (CCR) time measured on the first (CCR1) and sixth (CCR6) day of the adult butterfly stage

Final model	CCR1			CCR6		
	χ^2^	d.f.	*P*	χ^2^	d.f.	*P*
Developmental treatment	18.9	1	**<0.0001**	–	–	–
Adult treatment	–	–	–	6.2	1	**0.013**
Sex	2.8	1	0.09	2.5	1	0.11
Pupal weight	1.0	1	0.31	2.3	1	0.13
*T*_START_	0.005	1	0.95	0.8	1	0.37

The full linear mixed effect models included the fixed effects developmental treatment, adult treatment (only the CCR6 model) sex, pupal weight and temperature at the beginning of the experiment (*T*_START_), as well as interactions. On the basis of Akaike's information criterion (AIC) scores, the interaction terms were removed from both the CCR1 and CCR6 model, as well as the developmental treatment effect from the CCR6 model. *P*‐values in bold are significant.

Chill coma recovery time at day 6 of adult life (CCR6) was significantly influenced by the adult thermal treatment only (*P *=* *0.013, Table 2). This time, however, the individuals that had experienced cooler thermal conditions during their adulthood recovered faster than individuals from the warm adult treatment, with an average difference of 45 s, or 12% (Table 1 and Fig. 1). We also tested for the effect of developmental treatment and of the interaction between the developmental and adult treatment on CCR6, but both were nonsignificant (χ^2^ = 0.76, d.f. = 1, *P *=* *0.38 and χ^2^ = 0.31, d.f. = 1, *P *=* *0.58, respectively). The model without any interaction terms and without developmental treatment as a factor had the best fit with the lowest AIC score. Again, sex, pupal weight or *T*_START_ did not significantly impact CCR (Table 2). The random family effect explained 12% of the total variance (family variance = 0.0016 ± 0.040 SD; residual variance = 0.012).

The lack of sex‐specific CCR responses in our experiment may potentially be due to the relatively small female sample size. The unbalanced sex ratio results from rearing the Glanville fritillary in the laboratory under controlled conditions. After ‘waking up’ from the diapause as small larvae, the female caterpillars often return to diapause after a period of larval development.

### Genotype–phenotype association

**Figure 2:**
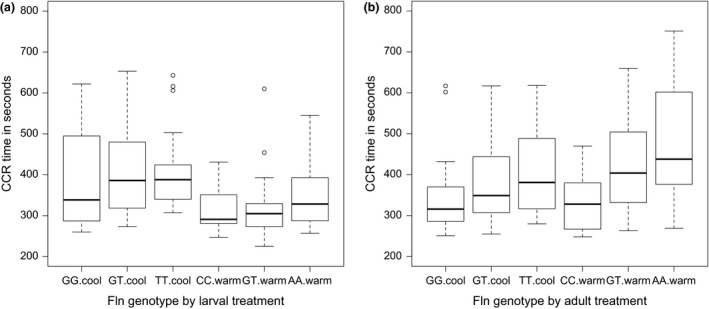
(a) The effects of *flightin* genotypes on chill coma recovery (CCR) time (in seconds) measured on the first day of adult life, for the two larval treatment groups (cool and warm), and (b) the effects of *flightin* genotypes on CCR time (in seconds) measured on the 6th day of adult life, for the two adult treatment groups (cool and warm). The lower box indicates the second quartile of values and the upper box the third quartile, the belt shows the median, and the whiskers represent the 1.5× interquartile range of the lower and upper quartiles, with outliers beyond the whiskers.

We associated 15 SNPs in nine genes with log_10_ CCR time. The SNP in the *flightin* gene showed a significant association with CCR (allelic effect size A>C = 0.076, χ^2^ = 9.9, d.f. = 1, *P *=* *0.0017), which remained significant after applying Bonferroni adjustment for multiple testing (corrected for 9 genes, threshold *P*_corrected_ = 0.0056). After the cold developmental treatment, AA genotypes took on average 18 s (5%) longer than CC genotypes to recover from chill coma, whereas after the warm developmental treatment AA genotypes took 34 s (11%) longer than TT genotypes. The difference in CCR times between genotypes was more pronounced in the second CCR measurement: in butterflies from the cold adult treatment, AA genotypes were 47 s (13%) slower than CC genotypes from the same treatment, whereas the AA genotypes were 143 s (42%) slower than CC genotypes for butterflies from the warm adult treatment. Figure 2 presents the CCR times for the *flightin* genotypes per treatment and CCR measurement, showing an additive (co‐dominant) effect of the alleles on CCR time. None of the other SNPs had a significant effect on CCR. The complete list of genotype–phenotype associations including allele effect sizes is given in Table S2.

Without more extensive sequence data, we cannot exclude the possibility of the family structure in the samples affecting the *flightin* genetic association result through LD with other genes. However, we believe this is likely not the case in our study. Firstly, only *flightin* and none of the other genes showed a genetic association with CCR. This is not due to the other SNPs occurring at lower frequencies, as the SNPs generally did not have low minor allele frequencies (Table S2). Also, the SNP alleles were generally well distributed across the families, which is evident from the relatively low variance of allele frequencies across families (Table S2). Furthermore, the percentage of variance explained by the random factor family was nearly the same in the model without SNPs (8.5%, family variance = 0.0011 ± 0.0329 SD, total variance = 0.0129) as in the model including the *flightin* SNP (8.8%, family variance = 0.0011 ± 0.0327 SD, total variance = 0.0125).

## Discussion

### Developmental plasticity and adult acclimation of cold tolerance

Plastic responses to thermal conditions can be a vital adaptation to seasonal or daily temperature variation by increasing an organism's physiological tolerance to thermal stress (Angilletta, 2009). For instance, it has been shown that exposure to colder temperatures during development or the adult stage can result in an increased cold tolerance in various animals, such as insects (Overgaard *et al*., 2011), fish (Fangue *et al*., 2006) and amphibians (McCann *et al*., 2014). Demonstrated adaptive advantages of such developmental or adult plasticity in response to thermal conditions include increased survival (Rako & Hoffmann, 2006), faster locomotion (Deere & Chown, 2006) and more successful foraging (Kristensen *et al*., 2008). However, an acclimated phenotype that is beneficial in a particular environment can come at a fitness cost when it is expressed in a mismatched environment (Kristensen *et al*., 2008). There are also many examples of nonadaptive plastic responses to thermal conditions that do not lead to enhanced performance or fitness (Leroi *et al*., 1994; Deere & Chown, 2006).

One of the necessary conditions for the evolution of adaptive plasticity is that the environment provides reliable cues predicting future conditions (Via *et al*., 1995). When plasticity is advantageous at a later life stage rather than immediately, it is a predictive adaptive response. Theoretical work suggests that this type of plasticity is less likely to evolve when the relationship between the cue and the late‐life environment weakens (Reed *et al*., 2010; but see, e.g. Sultan & Spencer, 2002). We did not find any evidence for a predictive adaptive response to thermal conditions during development in the Glanville fritillary butterfly. Instead, individuals that experienced cooler thermal conditions during development took on average 25% longer to recover from the cold shock at the first day of the adult stage (Fig. 1 and Table 1), indicating a physiological constraint of growth under cooler temperatures. Cold‐developed butterflies had a larger body size, which is consistent with a frequently observed pattern in other species (Temperature Size Rule, Atkinson, 1994), but this did not account for the difference in recovery time between treatments (Tables 1 and 2). By contrast, the adult acclimation treatment affected the CCR response as would be predicted for an adaptive plasticity response: butterflies that experienced cooler conditions during the adult stage recovered faster from the cool treatment. The cold recovery at this stage was not affected by thermal conditions during the developmental stage (Fig. 1 and Table 2). Our results thus show opposing effects of thermal conditions on plasticity during different life stages. A possible explanation could be that the capacity for plasticity is limited during development because of resource allocation to rapid development. For reviews on the physiology of insect cold tolerance plasticity, see, e.g. Sinclair & Roberts (2005), Teets & Denlinger (2013).

**Figure 3:**
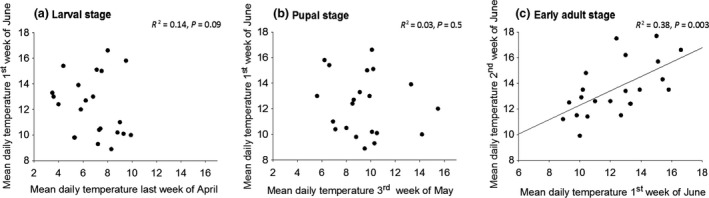
Regression of average daily temperatures on the Åland Islands (Jomala airport) between (a) late April and early June, (b) third week of May and early June, and (c) early June and mid‐June during 1993–2013.

The evolution of an adaptive plastic response of adult cold tolerance to thermal conditions during the adult stage, but not the developmental stage, makes sense when considering the ecology of the Glanville fritillary. On the Åland Islands, the thermal conditions experienced by the larvae or pupae do not generally predict those experienced by adults. Based on 21 years (1993–2013) of weather data (Jomala weather station, Finnish Meteorological Institute) from the Åland Islands, the average daily temperature experienced by the larvae during final stages of their development (end of April), or during the pupal stage (May), is not significantly correlated with the thermal conditions at the onset of the adult stage in early June (Fig. 3a,b). By contrast, the temperature during the first week of the adult stage in the wild is significantly correlated with that of the second week, explaining 38% of the variation (Fig. 3c). This means that later adult thermal conditions can (on average) be predicted from early adult conditions, allowing the butterflies to adjust their physiology adaptively. However, ongoing climate change is causing increased unpredictability of temperature variation, which may lead to a decrease in adaptive value of adult thermal acclimation, and consequently a decline in population fitness, in our study system.

### Genotypic effects on cold tolerance

The presence of genetic variation underlying cold tolerance has been demonstrated in among‐population and among‐species comparisons under common garden conditions, in particular in *Drosophila* species (Hoffmann *et al*., 2002; Ayrinhac *et al*., 2004). In *Drosophila melanogaster*, CCR is highly heritable (Anderson *et al*., 2005) and responds rapidly to artificial selection (Anderson *et al*., 2005; Bertoli *et al*., 2010). However, the molecular genetic basis of thermal tolerance is still largely unknown, especially for insects other than *Drosophila* (but see, e.g. de Jong *et al*., 2013; Franke *et al*., 2012; Karl *et al*., 2008 for examples in butterflies).

Our analysis revealed a significant association between CCR and a SNP in the gene *flightin*. Figure 2 shows a pattern consistent with an additive allelic effect for both CCR measurements (on the first and sixth day of adult life) and in both temperature treatment groups, which is particularly clear in the late adult CCR measurement. *Flightin*, initially identified in *D. melanogaster* (Vigoreaux *et al*., 1993), is a filament protein that plays a key role in indirect flight muscle (IFM) function, including its contractile activity (Ayer & Vigoreaux, 2003). In *D. melanogaster* and many other insects, the IFMs are the primary power source of flight (Josephson, 2006). Whereas most research on *flightin* has involved *D. melanogaster*, Xue *et al*. (2013) recently demonstrated that the gene is conserved across the Pancrustaceans. For example, in the brown planthopper, *Nilaparvata lugens*,* flightin* drives wing movement as well as vibration of the male‐specific tymbal (Xue *et al*., 2013). To our knowledge, our study is the first to show an association between *flightin* genetic variation and cold tolerance, or thermal adaptation in general.

In our experiment, CCR time was measured as the time it took the butterfly to recover from lying on its side to standing back on its feet, which was partly achieved by moving or beating the wings. The key role of *flightin* in insect flight muscle function therefore clearly suggests that the gene is functionally involved in the observed variation in CCR. The physiological mechanisms underlying insect chill coma are not yet well understood but most likely involve failure of nerves to generate action potentials or of muscles to achieve contractions (possible physiological mechanisms reviewed in Macmillan & Sinclair, 2011; Overgaard & MacMillan, 2017). In our study, the SNP in *flightin* codes for a nonsynonymous substitution in the coding region, leading to an amino acid change between Alanine (Ala) and Glutamic Acid (Glu), which have distinct chemical properties. An interesting next step would be to explore the functional link between the *flightin* SNP and CCR, for example, by measure the excitability of Glanville fritillary IFMs at a range of low temperatures and test for an association with *flightin*.

*Flightin* was included here based on results of a previous gene expression study in the Glanville fritillary, where it showed significantly higher expression during the final stages of larval development under cool compared to warm thermal conditions (Kvist *et al*., 2013), further indicating a role of this gene in thermal adaptation. Several recent Glanville fritillary studies have included *flightin* as a candidate gene to test for associations with performance or fitness traits. Mattila (2015) found a weak association of *flightin* with flight metabolic rate (which is related to flight activity and dispersal in the Glanville fritillary) in male butterflies. However, in a study measuring flight performance 24 h after exposure to different thermal conditions (15, 24 and 35 °C) by Wong *et al*. (2016), no effect of *flightin* on flight metabolic rate or interaction with temperature was found. There was also no effect of *flightin* in a study assessing reproductive performance of individuals under semi‐natural outdoor conditions (de Jong *et al*., 2014), or in a study testing for outlier loci in relation to habitat fragmentation (Fountain *et al*., 2016), but these studies were not designed to test for the effects of temperature. A possible explanation for the general lack of an effect of *flightin* in these studies may be that none of them assessed performance or fitness traits involving flight or locomotion at low temperatures, at which the *flightin* polymorphism may have an effect.

We did not find significant associations of CCR with SNPs in the other candidate genes in our study. A number of previous studies on the Glanville fritillary have revealed an association of a polymorphism in the gene *Pgi* with flight metabolism, dispersal and other life history traits through variation in thermal performance (reviewed in Niitepõld & Saastamoinen, 2017), but these studies did not involve recovery from subzero temperatures. Saastamoinen & Hanski (2008) showed that females with a specific *Pgi* genotype were able to fly at lower ambient temperatures and consequently could initiate oviposition earlier in the afternoon, when the environmental conditions are most favourable and the average egg clutch size is generally largest. A similar experimental approach where individual Glanville fritillary performance involving the IFMs (e.g. flight, or other locomotion or behaviour involving wing movements) is assessed under a wider thermal range including low temperatures will be an interesting focus of future research to investigate the role of *flightin* in performance and fitness of the Glanville fritillary.

## Conclusions

Here we have shown that plasticity can have very different effects on adult cold tolerance in the Glanville fritillary butterfly, depending on the life stage during which changes in thermal condition are experienced. In our study, adult acclimation led to an adaptive change in cold tolerance, in contrast to a nonadaptive plastic response to thermal conditions during late development. Taking into account the ecological conditions of the Glanville fritillary in Finland, these results support the theoretical prediction that adaptive plasticity evolves only when the environment provides reliable cues for future conditions. Our results also indicate – for the first time – a role of the *flightin* gene in cold tolerance, and that both phenotypic plasticity and genetic variation can have substantial effects on cold tolerance in our study system.

## Data accessibility

- Gene and SNP information: uploaded as online supporting information (Table S1, Appendix S1).
- Complete genotype–phenotype association results: uploaded as online supporting information (Table S2).
- Phenotypic and genotypic data: DRYAD: https://doi.org/10.5061/dryad.h14r5

## Supporting information


**Table S1** Candidate gene and SNP information.

**Table S2** Full genotype‐phenotype association results.
**Appendix S1** Full reference list for Table S1.
