# Supplementary material for: Environmental and genetic control of cold tolerance in the Glanville fritillary butterfly
Source: J Evol Biol. 2018 Mar 3;31(5):636–45. doi: 10.1111/jeb.13247 (PMC5969317; doi:10.1111/jeb.13247)
Supplement: Supplementary file 1 — Table S1 Candidate gene and SNP information. [file JEB-31-636-s001.docx]

Table S1: Candidate gene and SNP information

| **Gene name** | **Short gene name** | **Relevant gene function in insects** | **References** | **Relevant M. cinxia data** | **M. cinxia references** | **Gene ID in Ensembl Metazoa** | **Scaffold** | **Position in scaffold** | **Alleles** | **Nature of SNP change** | **Excluded from analysis** | **Reason for exclusion** |
| --- | --- | --- | --- | --- | --- | --- | --- | --- | --- | --- | --- | --- |
| c-Jun N-terminal protein kinase1 | JNK | Protein kinase, stress responses, role in longevity and immunity | Wang et al. 2005,  Boutros et al. 2002,  Wojda et al. 2004 | EST data comparing within and between population allele frequency variation | Vera et al. 2008 | MCINX008971 | scaffold 3121 | 13208 | A-G | non-synonymous | YES | no variation |
| Cytochrome P450 337 | Cyp337_a | Metabolic enzyme, metabolism of host plant chemicals and other xenobiotics | Berenbaum 2002,  Li et al. 2007,  Feyereisen et al. 2012 | Gene expression differences between populations, families and experimental treatments | Kvist et al. 2013,  de Jong et al. 2014 | MCINX011658 | scaffold 4593 | 27810 | A-G | synonymous | - |  |
| Cytochrome P450 337 | Cyp337_b | Metabolic enzyme, metabolism of host plant chemicals and other xenobiotics | Berenbaum 2002,  Li et al. 2007,  Feyereisen et al. 2012 | Gene expression differences between populations, families and experimental treatments | Kvist et al. 2013,  de Jong et al. 2014 | MCINX011658 | scaffold 4593 | 27912 | A-G | synonymous | - |  |
| Cytochrome P450 337 | Cyp337_c | Metabolic enzyme, metabolism of host plant chemicals and other xenobiotics | Berenbaum 2002,  Li et al. 2007,  Feyereisen et al. 2012 | Gene expression differences between populations, families and experimental treatments | Kvist et al. 2013,  de Jong et al. 2014 | MCINX011658 | scaffold 4593 | 27735 | C-T | synonymous | - |  |
| Flightin | fln | Flight muscle protein, affects flight muscle physiology and metabolism, and flight performance | Barton et al. 2005,  Contompasis 2010 | Gene expression differences between populations, families and experimental treatments | Kvist et al. 2013 | MCINX003215 | scaffold 1687 | 14486 | C-A | non-synonymous | - |  |
| Glucose-6-phosphate 1-dehydrogenase | G6pd_a | Catalytic enzyme in glycolytic pathway (closely linked with Pgi) and Krebs cycle. Part of in central metabolism | Eanes et al. 2006,  Flowers et al. 2007,  Eanes 2011 | EST data comparing within and between population allele frequency variation | Vera et al. 2008 | N/A | scaffold 470 | 36948 | G-T | non-synonymous | - |  |
| Glucose-6-phosphate 1-dehydrogenase | G6pd_b | Catalytic enzyme in glycolytic pathway (closely linked with Pgi) and Krebs cycle. Part of central metabolism | Eanes et al. 2006,  Flowers et al. 2007,  Eanes 2011 | EST data comparing within and between population allele frequency variation | Vera et al. 2008 | N/A | scaffold 470 | 37074 | A-C | non-synonymous | - |  |
| Heat shock 70 kDa protein 1 | Hsp70 1_a | Heat shock protein, environmental stress responses, interaction with Pgi | Rank et al. 2007,  Dahlhoff & Rank 2000,  Karl et al. 2009 | Gene expression linked to Pgi genotype and expression | Luo et al. 2014 | MCINX002266 | scaffold 1524 | 25279 | C-T | synonymous | YES | failed HWE test |
| Heat shock 70 kDa protein 1 | Hsp70 1_a | Heat shock protein, environmental stress responses, interaction with Pgi | Rank et al. 2007,  Dahlhoff & Rank 2000,  Karl et al. 2009 | Gene expression linked to Pgi genotype and expression. | Luo et al. 2014 | MCINX002266 | scaffold 1524 | 25351 | G-T | synonymous | YES | failed HWE test |
| Heat shock 70 kDa protein 2 | Hsp70 2 | Heat shock protein, environmental stress responses, interaction with Pgi | Rank et al. 2007,  Dahlhoff & Rank 2000,  Karl et al. 2009 | Gene expression linked to Pgi genotype and expression. | Luo et al. 2014 | MCINX009787 | scaffold 4593 | 72958 | C-T | synonymous | - |  |
| Heat shock 70 kDa protein 4 | Hsp70 4_a | Heat shock protein, environmental stress responses, interaction with Pgi | Rank et al. 2007,  Dahlhoff & Rank 2000,  Karl et al. 2009 | Gene expression linked to Pgi genotype and expression. | Luo et al. 2014 | MCINX003735 | scaffold 1785 | 58421 | A-G | synonymous | - |  |
| Heat shock 70 kDa protein 4 | Hsp70 4_b | Heat shock protein, environmental stress responses, interaction with Pgi | Rank et al. 2007,  Dahlhoff & Rank 2000,  Karl et al. 2009 | Gene expression linked to Pgi genotype and expression. | Luo et al. 2014 | MCINX003735 | scaffold 1785 | 58523 | A-G | synonymous | - |  |
| **Gene name** | **Short gene name** | **Relevant gene function in insects** | **References** | **Relevant M. cinxia data** | ***M. cinxia* references** | **Gene ID in Ensembl Metazoa** | **Scaffold** | **Position in scaffold** | **SNPs** | **Nature of SNP change** | **Excluded from analysis** | **Reason for exclusion** |
| Peripheral-type benzodiazepine receptor | PBR | Translocator protein, affects longevity and ageing | Curtis et al. 2007,  Tonoki et al. 2009 | Gene expression divergence between populations, families and/or experimental treatments | Kvist et al. 2013 | MCINX002817 | scaffold 1612 | 162678 | A-G | non-synonymous | - |  |
| Phosphoglucose isomerase | Pgi_a | Catalytic enzyme in glycolytic pathway. Role in central metabolism and energy production | Wheat 2010 | Pgi genotype and gene expression linked to physiological traits (e.g. flight metabolic rate and performance), life history traits (e.g. body size, fecundity) and population growth | Orsini et al. 2008,  Niitepõld et al. 2009, Saastamoinen et al. 2009,  Hanski 2011, Wong et al. 2016 | MCINX009374 | scaffold 3283 | 18361 | A-T | non-synonymous | - |  |
| Phosphoglucose isomerase | Pgi_b | Catalytic enzyme in glycolytic pathway. Role in central metabolism and energy production | Wheat 2010 | Pgi genotype and gene expression linked to physiological traits (e.g. flight metabolic rate and performance), life history traits (e.g. body size, fecundity) and population growth | Orsini et al. 2008,  Niitepõld et al. 2009, Saastamoinen et al. 2009,  Hanski 2011,  Wong et al. 2016 | MCINX009374 | scaffold 3283 | 24287 | A-G | non-synonymous | - |  |
| Phosphoglucose isomerase | Pgi_c | Catalytic enzyme in glycolytic pathway. Role in central metabolism and energy production | Wheat 2010 | Pgi genotype and gene expression linked to physiological traits (e.g. flight metabolic rate and performance), life history traits (e.g. body size, fecundity) and population growth | Orsini et al. 2008,  Niitepõld et al. 2009, Saastamoinen et al. 2009,  Hanski 2011,  Wong et al. 2016 | MCINX009374 | scaffold 3283 | 19949 | A-C | non-synonymous | - |  |
| Succinate Dehydrogenase complex subunit D | SDHD | Subunit of enzyme active in Krebs cycle and electron transport chain. Part of central metabolism and energy production. Linked to flight performance and population growth | Wheat et al. 2011,  Klepsatel & Flatt 2011,  Marden et al. 2013 | Gene expression differences between old and new populations, genotype linked to flight metabolic rate and population growth. Interaction with Pgi and other carbohydrate metabolic genes | Wheat et al. 2011,  Klepsatel & Flatt 2011,  Marden et al. 2013 | MCINX007660 | scaffold 266 | 99376 | A-G | synonymous | - |  |
| Troponin-T | TnT | Muscle protein, role in flight muscle energy metabolism and flight performance | Marden et al. 1999,  Marden et al. 2008 | Gene expression divergence between populations, families and/or experimental treatments | Kvist et al. 2013 | MCINX001487 | scaffold 1353 | 15434 | C-T | non-synonymous | - |  |

The candidate genes and SNPs were selected on the basis of their known function in insect physiology and on previous Glanville fritillary studies, summarised here. Gene model IDs, scaffolds, and positions on scaffolds refer to the published *M. cinxia* genome in Ensembl Metazoa (Ahola et al. 2014). The small letter after the short gene name is used when multiple SNPs for the gene were used. For full references see supplementary material Appendix S1.
