# Supplementary material for: Environmental and genetic control of cold tolerance in the Glanville fritillary butterfly
Source: J Evol Biol. 2018 Mar 3;31(5):636–45. doi: 10.1111/jeb.13247 (PMC5969317; doi:10.1111/jeb.13247)
Supplement: Supplementary file 2 — Table S2 Full genotype‐phenotype association results. Appendix S1 Full reference list for Table S1. [file JEB-31-636-s002.docx]

**Table S2: Full results genotype-phenotype association with log_10_ CCR time**

| **Gene name** | **Gene ID** | **SNP position** | **SNP change** | **Allele** | **Allele freq.** | **Allele freq. variance families** | **Effect size** | **X^2^** | ***P*** |
| --- | --- | --- | --- | --- | --- | --- | --- | --- | --- |
| Cyp337_a | MCINX011658 | 4593:27810 | A>G | A | 0.54 | 0.10 | -0.012 | 0.11 | 0.74 |
| Cyp337_b | MCINX011658 | 4593:27912 | A>G | A | 0.54 | 0.10 | -0.012 | 0.11 | 0.74 |
| Cyp337_c | MCINX011658 | 4593:27735 | C>T | C | 0.54 | 0.10 | -0.012 | 0.11 | 0.74 |
| Fln | MCINX003215 | 1687:14486 | C>A | A | 0.59 | 0.10 | 0.076 | 9.88 | **0.0017** |
| G6pd_a | NA | 470:36948 | G>T | G | 0.16 | 0.04 | 0.039 | 1.22 | 0.27 |
| G6pd_b | NA | 470:37074 | A>C | C | 0.09 | 0.02 | 0.063 | 1.46 | 0.23 |
| Hsp70.2 | MCINX009787 | 4593:72958 | C>T | T | 0.23 | 0.04 | 0.047 | 1.67 | 0.20 |
| Hsp70.4_a | MCINX003735 | 1785:58421 | A>G | G | 0.51 | 0.04 | -0.008 | 0.10 | 0.76 |
| Hsp70.4_b | MCINX003735 | 1785:58523 | A>G | A | 0.51 | 0.04 | -0.008 | 0.10 | 0.76 |
| PBR | MCINX002817 | 1612:162678 | A>G | A | 0.37 | 0.09 | 0.007 | 6.17 | 0.01 |
| Pgi_a | MCINX009374 | 3283:18361 | A>T | A | 0.51 | 0.05 | -0.012 | 0.20 | 0.65 |
| Pgi_b | MCINX009374 | 3283:24287 | A>G | G | 0.58 | 0.09 | 0.000 | 0.00 | 1.00 |
| Pgi_c | MCINX009374 | 3283:19949 | A>C | C | 0.17 | 0.08 | -0.009 | 0.07 | 0.78 |
| SDHD | MCINX007660 | 266:99376 | A>G | A | 0.73 | 0.04 | 0.006 | 0.01 | 0.93 |
| TnT | MCINX001487 | 1353:15434 | C>T | T | 0.62 | 0.13 | -0.039 | 1.97 | 0.16 |

Allelic effect sizes, allele frequencies, allele frequency variance across families, allelic effect size, and likelihood ratio test results for the genotype-phenotype association analysis of the candidate SNPs with log_10_ chill coma recovery (CCR) time. For more gene and SNP information see Table S1.

**Appendix S1: Full reference list for Table S1**

Ahola V, Lehtonen R, Somervuo P, Salmela L, Koskinen P, Rastas P et al. (2014). The Glanville fritillary genome retains an ancient karyotype and reveals selective chromosomal fusions in Lepidoptera. *Nature communications*, **5**, 4737.

Barton B, Ayer G, Heymann N, Maughan DW, Lehmann FO, Vigoreaux JO (2005) Flight muscle properties and aerodynamic performance of Drosophila expressing a flightin transgene. *Journal of Experimental Biology* **208**, 549-560.

Berenbaum M (2002) Postgenomic chemical ecology: from genetic code to ecological intercations. *Journal of Chemical Ecology* **28**, 873-896.

Boutros M, Agaisse H, Perrimon N (2002) Sequential activation of signaling pathways during innate immune responses in Drosophila. *Developmental Cell* **3,** 711-722.

Contompasis JL, Nyland LR, Maughan DW, Vigoreaux JO (2010) Flightin Is Necessary for Length Determination, Structural Integrity, and Large Bending Stiffness of Insect Flight Muscle Thick Filaments. *Journal of Molecular Biology* **395**, 340-348.

Curtis C, Landis GN, Folk D, Wehr NB, Hoe N, Waskar M, Tower J (2007) Transcriptional profiling of MnSOD-mediated lifespan extension in Drosophila reveals a species-general network of aging and metabolic genes. *Genome biology* **8**, R262.

Dahlhoff EP, Rank NE (2000) Functional and physiological consequences of genetic variation at phosphoglucose isomerase: Heat shock protein expression is related to enzyme genotype in a montane beetle. *Proceedings of the National Academy of Sciences* **97**, 10056–10061.

de Jong, M.A., Wong, S.C., Lehtonen, R. & Hanski, I. (2014) Cytochrome P450 gene CYP337 and heritability of fitness traits in the Glanville fritillary butterfly. *Molecular Ecology,* **23,** 1994-2005.

Eanes WF (2011) Molecular population genetics and selection in the glycolytic pathway. *Journal of Experimental Biology* **214**,165-171.

Eanes WF, Merritt TJS, Flowers JM, Kumagai S, Sezgin E, Zhu C-T (2006) Flux control and excess capacity in the enzymes of glycolysis and their relationship to flight metabolism in Drosophila melanogaster. *Proceedings of the National Academy of Sciences* **103**, 19413-19418.

Feyereisen R (2012) Insect CYP genes and P450 enzymes. In: *Insect Molecular Biology and Biochemistry* (ed. Gilbert LI), pp. 236-316. Academic Press.

Flowers JM, Sezgin E, Kumagai S, Duvernell DD, Matzkin LM, Schmidt PS, Eanes WF (2007) Adaptive evolution of metabolic pathways in Drosophila. Molecular Biology and Evolution **24**,1347-1354.

Hanski IA (2011) Eco-evolutionary spatial dynamics in the Glanville fritillary butterfly. *Proceedings of the National Academy of Sciences* **108**, 14397-14404.

Karl I, Schmitt T. Fischer K (2008) Phosphoglucose isomerase genotype affects life-history traits and cold stress resistance in a Copper butterfly. Functional Ecology, **22**, 887–894.

Karl I, Sørensen JG, Loeschcke V, Fischer K (2009) Hsp70 expression in the Copper butterfly Lycaena tityrus across altitudes and temperatures. *Journal of Evolutionary Biology* **22**, 172–178.

Klepsatel P, Flatt T (2011) The genomic and physiological basis of life history variation in a butterfly metapopulation. *Molecular Ecology* **20**, 1795-1798.

Kvist J, Wheat CW, Kallioniemi E*, et al.* (2013) Temperature treatments during larval development reveal extensive heritable and plastic variation in gene expression and life history traits. *Molecular Ecology* **22**, 602-619.

Li X, Schuler MA, Berenbaum MR (2007) Molecular mechanisms of metabolic resistance to synthetic and natural xenobiotics. *Annual Reviews of Entomology* **52**, 231-253.

Luo, S., Chong Wong, S., Xu, C., Hanski, I., Wang, R. & Lehtonen, R. (2014) Phenotypic plasticity in thermal tolerance in the Glanville fritillary butterfly. *Journal of Thermal Biology,* **42,** 33-39.

Marden JH, Fitzhugh GH, Wolf MR, Arnold KD and Rowan B (1999) Alternative splicing, muscle calcium sensitivity, and the modulation of dragonfly flight performance. *Proceedings of the National Academy of Sciences* **96**,15304 -15309.

Marden JH, Fescemyer HW, Saastamoinen M, MacFarland SP, Vera JC, Frilander M, Hanski I (2008) Weight and nutrition affect pre-mRNA splicing of a muscle gene associated with performance, energetics and life history. *Journal of Experimental Biology* **211**, 3653-3660.

[Marden JH](http://www.ncbi.nlm.nih.gov/pubmed?term=Marden%20JH%5BAuthor%5D&cauthor=true&cauthor_uid=23550759), [Fescemyer HW](http://www.ncbi.nlm.nih.gov/pubmed?term=Fescemyer%20HW%5BAuthor%5D&cauthor=true&cauthor_uid=23550759), [Schilder RJ](http://www.ncbi.nlm.nih.gov/pubmed?term=Schilder%20RJ%5BAuthor%5D&cauthor=true&cauthor_uid=23550759), [Doerfler WR](http://www.ncbi.nlm.nih.gov/pubmed?term=Doerfler%20WR%5BAuthor%5D&cauthor=true&cauthor_uid=23550759), [Vera JC](http://www.ncbi.nlm.nih.gov/pubmed?term=Vera%20JC%5BAuthor%5D&cauthor=true&cauthor_uid=23550759), [Wheat CW](http://www.ncbi.nlm.nih.gov/pubmed?term=Wheat%20CW%5BAuthor%5D&cauthor=true&cauthor_uid=23550759) (2013) Genetic variation in HIF signaling underlies quantitative variation in physiological and life-history traits within lowland butterfly populations. *Evolution* **67**, 1105-1115.

Niitepõld K, Smith AD, Osborne JL*, et al.* (2009) Flight metabolic rate and Pgi genotype influence butterfly dispersal rate in the field. *Ecology* **90**, 2223-2232.

Orsini L, Wheat CW, Haag CR*, et al.* (2009) Fitness differences associated with Pgi SNP genotypes in the Glanville fritillary butterfly (Melitaea cinxia). *Journal of Evolutionary Biology* **22**, 367-375.

Rank NE, Bruce DA, McMillan DM, Barclay C, Dahlhoff EP (2007) Phosphoglucose isomerase genotype affects running speed and heat shock protein expression after exposure to extreme temperatures in a montane willow beetle. *Journal of Experimental Biology* **210**, 750-64.

Saastamoinen M, Ikonen S, Hanski I (2009) Significant effects of Pgi genotype and body reserves on lifespan in the Glanville fritillary butterfly. *Proceedings of the Royal Society B: Biological Sciences* **276**, 1313-1322.

Tonoki A, Kuranaga E, Tomioka T, Hamazaki J, Murata S, Tanaka K, Miura M (2009) Genetic evidence linking age-dependent attenuation of the 26S proteasome with the aging process. *Molecular and cellular biology* **29**, 1095-1106.

Vera JC, Wheat CW, Fescemyer HW*, et al.* (2008) Rapid transcriptome characterization for a nonmodel organism using 454 pyrosequencing. *Molecular Ecology* **17**, 1636-1647.

Wang M C, Bohmann D, Jasper H (2005) JNK extends life span and limits growth by antagonizing cellular and organism-wide responses to insulin signaling. *Cell* **121**, 115-125.

Wheat CW (2010) Phosphoglucose isomerase (Pgi) performance and fitness effects among Arthropods and its potential role as an adaptive marker in conservation genetics. *Conservation Genetics* **11**, 387-397.

Wheat CW, Fescemyer HW, Kvist J, Tas EVA, Vera JC, Frilander MJ, Marden JH (2011) Functional genomics of life history variation in a butterfly metapopulation. *Molecular Ecology* **20,** 1813-1828.

Wong SC, Oksanen A, Mattila AL, Lehtonen R, Niitepõld K & Hanski I (2016). Effects of ambient and preceding temperatures and metabolic genes on flight metabolism in the Glanville fritillary butterfly. *Journal of insect physiology*, **85**, 23-31.
